# Far-Infrared Irradiation Induces Time-Dependent Endothelial-Protective and Vascular Repair-Associated Molecular Remodeling in Human Femoral Artery Endothelial Cells

**DOI:** 10.3390/cells15151390

**Published:** 2026-07-31

**Authors:** Makoto Saito, Ayuko Kimura, Sanshiro Hanada, Yuriko Hayashi, Hirokazu Kimura

**Affiliations:** 1 Department of Health Science, Gunma Paz University Graduate School, Takasaki 370-0006, Gunma, Japan; ma-saito@paz.ac.jp (M.S.);; 2 Advanced Medical Science Research Center, Gunma Paz University, Takasaki 370-0006, Gunma, Japan

**Keywords:** far-infrared radiation, human femoral artery endothelial cells (HFAECs), endothelial protection, phosphoproteomics, vascular repair

## Abstract

Far-infrared radiation (FIR) may influence vascular endothelial function, but its time-dependent molecular effects in adult arterial endothelial cells remain incompletely defined. We investigated FIR-induced responses in human femoral artery endothelial cells (HFAECs) using transcriptomic, proteomic, and phosphoproteomic analyses with three biological replicates per condition. Transcriptomic profiling 3 h after 30 min of FIR irradiation identified 424 differentially expressed genes (210 upregulated and 214 downregulated) among 16,081 genes, including endothelial-protective, nitric oxide-related, oxidative-stress, and heat-shock response genes. Proteomic analysis identified 13 differentially abundant proteins immediately after irradiation and 23, 75, and 61 proteins at 6, 12, and 24 h, respectively. These temporal changes involved mitogen-activated protein kinase kinase/extracellular signal-regulated kinase signaling, vascular maturation, cytoskeletal organization, cell polarity, calcium/nitric oxide regulation, and stress adaptation. Phosphoproteomic analysis identified 12, 10, and 53 phosphorylation-related changes at 6, 12, and 24 h, respectively, indicating progression from actin and adhesion remodeling to junctional reorganization, focal adhesion maturation, and mechanosensing. Network analysis identified caveolin-1, β-catenin, and lamin B1 as hubs in a Rho guanosine triphosphatase-related module. Collectively, FIR induced a coordinated endothelial adaptive molecular response in HFAECs; functional studies are required to determine whether these changes enhance vascular repair.

## 1. Introduction

Far-infrared radiation (FIR) has attracted increasing interest as a physical stimulus that may influence cellular function, vascular function, oxidative-stress responses, inflammatory regulation, and endothelial homeostasis beyond its thermal effects [1,2]. FIR-based thermal therapy and FIR-emitting devices have been applied in areas such as peripheral circulation, vascular endothelial function, pain control, and wound healing; however, the molecular mechanisms underlying these biological effects remain incompletely understood [1,3]. In vascular endothelial cells, FIR has been reported to affect nitric oxide (NO) production, endothelial nitric oxide synthase (eNOS)/nitric oxide synthase 3 (NOS3) activity, oxidative-stress responses, inflammatory signaling, and angiogenesis-associated pathways [2,3,4,5,6]. Nevertheless, the cell type-specific and time-dependent molecular responses of endothelial cells to FIR irradiation remain insufficiently characterized.

Vascular endothelial cells play central roles in maintaining vascular homeostasis, vasodilation, anti-inflammatory regulation, antithrombotic activity, vascular permeability, angiogenesis, and vascular repair [7]. Endothelial dysfunction contributes to a broad range of clinically important disorders, including atherosclerosis, peripheral artery disease, diabetic vascular complications, chronic kidney disease-associated vascular injury, ischemic wounds, diabetic foot ulcers, and delayed wound healing [8,9]. These conditions are characterized by combined impairment of NO production, increased oxidative stress, chronic inflammation, abnormal cell adhesion, altered vascular permeability, microcirculatory dysfunction, and reduced vascular repair capacity [8,9]. Therefore, determining whether FIR irradiation induces endothelial protection, NO-related signaling, cytoskeletal remodeling, junction remodeling, mechanotransduction, and vascular repair-associated molecular responses in endothelial cells is important for understanding the medical relevance of FIR.

An important clinical application of FIR is its use in patients undergoing hemodialysis to support arteriovenous fistula (AVF) function. Clinical studies and evidence syntheses have investigated whether repeated FIR treatment improves vascular access blood flow, promotes AVF maturation, and prolongs unassisted patency, although the magnitude and consistency of the reported benefits vary across study populations and treatment protocols [10,11,12,13,14]. This vascular-access application further underscores the need to clarify the endothelial-protective, nitric oxide-related, and vascular-remodeling responses induced by FIR.

Vascular repair and wound healing require not only angiogenesis, but also endothelial cell survival, migration, cell–cell adhesion, barrier integrity, extracellular matrix remodeling, and stabilization of newly remodeled vascular structures [7,8,9,15]. In ischemic wounds and diabetic foot ulcers, impaired peripheral circulation, endothelial dysfunction, microvascular injury, persistent inflammation, and reduced tissue oxygen delivery interfere with tissue repair [8,9,15]. Thus, if FIR induces a protective and vascular repair-supportive molecular phenotype in endothelial cells, its potential biological significance would not be limited to stimulation of angiogenesis. Rather, FIR may contribute to improvement of the molecular environment required for peripheral vascular homeostasis, endothelial barrier stabilization, and tissue repair.

Endothelial cells dynamically regulate transcriptional programs, protein abundance, and phosphorylation-dependent signaling in response to flow, mechanical stress, hypoxia, inflammatory stimuli, and thermal or physical stimulation [7,16,17]. These responses involve multiple pathways and molecular regulators, including Krüppel-like factor 2 (KLF2), NOS3, heme oxygenase 1 (HMOX1), heat shock proteins, mitogen-activated protein kinase kinase/extracellular signal-regulated kinase (MEK/ERK) signaling, Rho guanosine triphosphatase (Rho GTPase) signaling, Wingless/integrated β-catenin (Wnt/β-catenin) signaling, transforming growth factor-β (TGF-β)/bone morphogenetic protein (BMP) signaling, Ca^2+^/NO signaling, cytoskeletal remodeling, junction remodeling, and focal adhesion maturation [2,7,16,17,18]. KLF2 and NOS3 are closely linked to flow-responsive, anti-inflammatory, antithrombotic, and NO-dependent vascular protective phenotypes in arterial endothelial cells [16,18]. Caveolin 1 (CAV1), Rho GTPases, catenin beta 1 (CTNNB1), and focal adhesion-related molecules are involved in mechanotransduction, cell adhesion, cytoskeletal remodeling, and stabilization of vascular architecture [7,17]. Therefore, understanding the endothelial response to FIR irradiation requires an integrated analysis of time-dependent multilayered molecular changes rather than evaluation of a single molecule or pathway.

Previous studies on FIR-related endothelial responses have frequently used human umbilical vein endothelial cells (HUVECs) or human microvascular endothelial cells. These studies have suggested that FIR may modulate eNOS/NO production, MEK/ERK signaling, cell proliferation, cell migration, and angiogenesis-associated responses [4,5,6]. However, the angiogenic effects of FIR have not been fully resolved, because both pro-angiogenic and anti-angiogenic responses have been reported under different experimental conditions [5,6]. These apparently divergent findings may reflect differences in irradiation conditions, wavelength, intensity, exposure duration, thermal contribution, endothelial cell type, culture conditions, and time point of evaluation [1,5,6]. Therefore, the vascular endothelial effects of FIR should not be interpreted simply as pro-angiogenic or anti-angiogenic. Instead, FIR-induced responses should be analyzed according to endothelial cell type, temporal phase, and distinct biological processes, including endothelial protection, vascular repair, cytoskeletal remodeling, junction remodeling, and structural maturation.

HUVECs are derived from umbilical veins and retain developmental and angiogenic plasticity. Accordingly, findings obtained from HUVECs cannot necessarily be extrapolated directly to adult arterial endothelial cells. Adult arterial endothelial cells are particularly dependent on flow responsiveness, mechanical tension, anti-inflammatory and antithrombotic regulation, vascular wall homeostasis, barrier integrity, and structural maturation [7,16,17]. These functions are closely related to the pathophysiology of atherosclerosis, peripheral artery disease, ischemic vascular injury, and diabetic vascular complications [8,9]. Thus, analysis of FIR responses in adult arterial endothelial cells has important basic relevance for evaluating the potential clinical implications of FIR.

Human femoral artery endothelial cells (HFAECs) represent a useful model for evaluating molecular responses in adult arterial endothelial cells. The femoral artery is an important peripheral artery involved in lower-limb perfusion, ischemic vascular disease, atherosclerotic lesions, diabetic peripheral vascular complications, and impaired wound healing. Endothelial cells derived from this arterial bed may exhibit arterial-specific protective responses and structural remodeling programs. Therefore, clarifying how FIR irradiation affects transcriptional, proteomic, and phosphoproteomic responses in HFAECs is important for understanding the molecular basis of FIR-associated endothelial protection, peripheral vascular repair, ischemic tissue repair, and wound healing support.

In the present study, HFAECs were exposed to FIR irradiation, and time-dependent molecular responses were analyzed using DNA microarray, global proteomics, and global phosphoproteomics. Particular attention was given to changes immediately after FIR irradiation and at 6, 12, and 24 h after irradiation, thereby enabling integrated assessment of transcriptional changes, protein abundance changes, and phosphorylation-dependent signaling networks. The aim of this study was to determine whether FIR irradiation induces a time-dependent molecular program in HFAECs involving endothelial protection, MEK/ERK priming, vascular endothelial growth factor (VEGF)-noncentral angiogenesis-supportive responses, cytoskeletal remodeling, junction remodeling, Rho GTPase-related mechanotransduction, and structural maturation. Through this approach, we sought to clarify whether FIR may provide a molecular basis linking endothelial protection with vascular repair in adult arterial endothelial cells.

## 2. Materials and Methods

### 2.1. Cell Culture

HFAECs were purchased from Cell Applications Inc. (San Diego, CA, USA) as a Human Femoral Artery Endothelial Cells Total Kit and cultured in Human Endothelial Cell Growth Medium (CA213K500, Cell Applications Inc., San Diego, CA, USA), consisting of basal medium supplemented with growth additives including vascular endothelial growth factor (VEGF). Cells were maintained at 37 °C in a humidified incubator containing 5% CO_2_, and the culture medium was replaced every 2 days. Cells were passaged at approximately 70–80% confluence according to the manufacturer’s instructions and used for experiments at passage 3.

### 2.2. FIR Exposure

Far-infrared (FIR) irradiation was delivered using a ceramic FIR generator (WS^TM^ TY101F; WS Far Infrared Medical Technology, Taipei, Taiwan). This emitter generates electromagnetic waves with wavelengths ranging from 3 to 25 μm and a peak wavelength of 8.2 μm. HFAECs were seeded in 6-well culture plates (IWAKI, AGC Techno Glass Co., Ltd., Shizuoka, Japan) at a density of 1 × 10^6^ cells per well in 2 mL of culture medium and cultured prior to FIR exposure. Before irradiation, the culture medium was replaced and the lids of the culture plates were removed. The FIR emitter was positioned 20 cm above the bottom of the culture plates, and cells were exposed to FIR irradiation for 30 min. During irradiation, both the FIR-treated group and the negative control group, which was shielded with aluminum foil, were placed inside a Class II Type A/B3 biological safety cabinet (Panasonic Healthcare Co., Ltd., Osaka, Japan).Control cells were handled identically except that they were shielded from FIR irradiation by aluminum foil. After irradiation, the cells were either harvested immediately for subsequent analyses or further cultured at 37 °C in a humidified atmosphere containing 5% CO_2_ for additional experiments.

The present experiment was designed to characterize the temporal molecular response to one predefined FIR exposure condition (emission range, 3–25 μm; peak wavelength, 8.2 μm; emitter-to-plate distance, 20 cm; exposure duration, 30 min). No dose–response comparison across irradiation duration, emitter-to-sample distance, irradiance, or cumulative energy density was performed.

### 2.3. Transcriptomic Analysis

HFAECs were divided into non-irradiated control [FIR (-)] and FIR-irradiated groups [FIR (+)]. Cells in the FIR group were exposed to FIR for 30 min and subsequently cultured for an additional 3 h prior to RNA extraction. Three independent biological replicates were prepared for each experimental group (*n* = 3 per group).

#### 2.3.1. RNA Extraction

Total RNA was extracted from HFAECs using the RNeasy Mini Kit (QIAGEN, Hilden, Germany) according to the manufacturer’s instructions. Briefly, the culture medium was removed, and the cells were washed with phosphate-buffered saline (PBS). After complete aspiration of the residual buffer, the cells were lysed directly in the culture plates with Buffer RLT supplemented with β-mercaptoethanol. The lysates were collected and transferred to RNase-free microcentrifuge tubes. The lysates were homogenized using RNase-free pipette tips. An equal volume of 70% ethanol was then added to each homogenized lysate, and the samples were thoroughly mixed by pipetting without centrifugation. Up to 700 μL of each sample was applied to an RNeasy Mini spin column and centrifuged at ≥8000× *g* for 15 s. When the sample volume exceeded 700 μL, the remaining lysate was sequentially loaded onto the same spin column and centrifuged under identical conditions. To minimize genomic DNA contamination, on-column DNase digestion was performed using the RNase-Free DNase Set (QIAGEN, Hilden, Germany) according to the manufacturer’s instructions. The column membrane was subsequently washed with 700 μL of Buffer RW1, followed by two washes with 500 μL of Buffer RPE. The first wash was performed by centrifugation at ≥8000× *g* for 15 s, whereas the second wash was performed at ≥8000× *g* for 2 min to dry the membrane and minimize ethanol carryover. The spin column was then transferred to a new RNase-free 1.5 mL collection tube, and total RNA was eluted with 30 μL of RNase-free water by centrifugation at 12,000× *g* for 3 min. The purified RNA was stored at −80 °C until subsequent DNA microarray analysis.

#### 2.3.2. RNA Quality Assessment

RNA concentration was determined using the Qubit RNA HS Assay Kit and a Qubit fluorometer (Thermo Fisher Scientific, Waltham, MA, USA). RNA purity and integrity were evaluated by Filgen, Inc. (Aichi, Japan) using spectrophotometric analysis and an Agilent 2100 Bioanalyzer (Agilent Technologies, Santa Clara, CA, USA), respectively. Only RNA samples with an RNA integrity number (RIN) of ≥7.0 were used for DNA microarray analysis.

#### 2.3.3. DNA Microarray Analysis

RNA samples that met the quality criteria were submitted to Filgen, Inc. (Aichi, Japan) for transcriptomic analysis. Genome-wide gene expression profiling was performed using the Clariom™ S Human Assay (Thermo Fisher Scientific, Waltham, MA, USA) according to the manufacturer’s instructions. Briefly, total RNA was reverse-transcribed to generate complementary DNA (cDNA), followed by amplification and in vitro transcription to produce complementary RNA (cRNA). The purified cRNA was subsequently reverse-transcribed to synthesize single-stranded cDNA (ss-cDNA), which was fragmented and labeled using the GeneChip™ WT PLUS Reagent Kit (Thermo Fisher Scientific, Waltham, MA, USA). The labeled ss-cDNA was then hybridized to Clariom™ S Human Arrays, followed by washing, staining, and scanning according to the manufacturer’s standard protocols. All microarray procedures, including labeling, hybridization, scanning, and primary data processing, were performed by Filgen, Inc. Raw fluorescence intensity data (CEL files) were analyzed using Transcriptome Analysis Console (TAC) software (version 4.0.2; Thermo Fisher Scientific, Waltham, MA, USA). Probe-level data were background-corrected, normalized, and summarized using the Signal Space Transformation-Robust Multi-array Average (SST-RMA) algorithm [19]. The resulting gene expression values were used for subsequent analyses. Differentially expressed genes (DEGs) between FIR (-) and FIR (+) were identified using a threshold of fold-change > 1.5 or <−1.5 and a *p*-value < 0.05 determined by pairwise analysis and a statistical significance of *p*-value < 0.05. Differential gene expression patterns were visualized using volcano plots generated from normalized expression data.

#### 2.3.4. Functional Enrichment Analysis

Functional enrichment analysis of DEGs was performed using Metascape (version 3.5.20260701; https://metascape.org; accessed on 30 June 2026) [20]. Reactome Gene Sets, Kyoto Encyclopedia of Genes and Genomes (KEGG) pathways, and WikiPathways were included in the analysis [21,22,23]. Enriched terms with a Benjamini–Hochberg-adjusted *p*-value of <0.05 were considered statistically significant [24]. Redundant terms with substantial overlap were grouped, and representative pathways were selected based on their biological relevance to endothelial function, angiogenesis, inflammation, cellular stress responses, and cytoskeletal organization.

### 2.4. Proteomic and Phosphoproteomic Analyses

For Proteomic and phosphoproteomic analysis, cells were harvested immediately after FIR irradiation (0 h) and at 6, 12, and 24 h after irradiation. Differential protein expression at each time point was determined by comparison with the non-irradiated control group (0 h). Three independent biological replicates were analyzed for each condition.

#### 2.4.1. Protein Extraction, Digestion and Peptide Preparation

To obtain cell lysates, the cells were sonicated on ice ten times for 30 s each at 28 kHz using a BIORUPTOR-One sonicator (Sonic Bio Co., Ltd., Kanagawa, Japan) in denaturation buffer (20 mM HEPES [pH 8.0], 9 M urea) supplemented with phosphatase inhibitor cocktails 1 and 2 (Sigma-Aldrich, St. Louis, MO, USA). Cleared cell lysate was obtained by centrifugation at 15,000 *g* for 15 min at 4 °C. For each cell line, 50 μg of protein from the cleared cell lysate was reduced with 10 mM dithiothreitol and subsequently alkylated with 10 mM iodoacetamide. After dilution with 3 volumes of 20 mM HEPES, proteins were digested with 1:10 (enzyme/substrate *w*/*w*) Trypsin Platinum, Mass Spectrometry Grade (Promega Corporation, Madison, WI, USA) at 37 °C for 17 h. The digested mixture was acidified with 20% trifluoroacetic acid (TFA). Peptides were desalted using StageTips filled with C18 Empore disc membranes. After desalting with StageTips, peptides were evaporated in a vacuum concentrator, then dissolved in 0.1% TFA for the liquid chromatography–tandem mass spectrometry (LC–MS/MS) analysis.

#### 2.4.2. Phosphopeptide Enrichment

For phosphoproteomic analysis, approximately 50 μg of tryptic peptides was subjected to phosphopeptide enrichment using the Titanosphere Phos-TiO Kit (GL Sciences, Tokyo, Japan) according to the manufacturer’s instructions and the aliphatic hydroxy acid-modified metal oxide chromatography (HAMMOC) method [25]. Briefly, peptides were acidified and loaded onto TiO_2_ beads to selectively capture phosphorylated peptides. After extensive washing to remove non-phosphorylated peptides, phosphopeptides were eluted according to the manufacturer’s protocol, desalted using C18 StageTips, and dried in a vacuum concentrator. The dried phosphopeptides were reconstituted in 0.1% TFA prior to LC-MS/MS analysis.

#### 2.4.3. LC–MS/MS Analysis

Mass spectrometric analysis was performed using a Vanquish Neo ultra-high-performance liquid chromatography system (Thermo Fisher Scientific, Waltham, MA, USA) coupled to an Orbitrap Exploris 240 mass spectrometer (Thermo Fisher Scientific, Waltham, MA, USA), a quadrupole-Orbitrap Fourier transform mass spectrometer. Data acquisition and instrument control were performed automatically using Xcalibur software (version 4.7.102.25; Thermo Fisher Scientific, Waltham, MA, USA). Peptide samples were first trapped and concentrated online on a reversed-phase precolumn (PepMap™ Neo Trap Cartridge; Thermo Fisher Scientific) connected to the ultra-high-performance liquid chromatography system. The trapped peptides were subsequently separated on a 3-μm nano-high-performance liquid chromatography capillary reversed-phase analytical column (Nikkyo Technos, Tokyo, Japan) using an acetonitrile (ACN) gradient. Mobile phase A consisted of 0.1% (*v*/*v*) fluoroacetic acid (FAA) and 2% (*v*/*v*) ACN, whereas mobile phase B consisted of 0.1% (*v*/*v*) FAA and 95% (*v*/*v*) ACN. Peptides were eluted at a flow rate of 300 nL/min. Peptides were ionized in positive ion mode by electrospray ionization and introduced into the mass spectrometer. Prior to the analytical runs, a preliminary analysis was performed using one-hundredth of the sample amount with a 20 min gradient to evaluate peptide peak intensities. Based on the results of this preliminary analysis, the sample loading amount was adjusted so that the maximum peptide peak intensity did not exceed 1 × 10^9^. The main mass spectrometric analysis was subsequently performed using a 135 min gradient program consisting of 0–121 min (6–31% B), 121–126 min (31–50% B), 126–130 min (50–90% B), and 130–135 min (90% B). Full MS spectra (MS1) were acquired at a resolution of 60,000 over an m/z range of 375–1500 m/z. MS/MS spectra were acquired at a resolution of 15,000 using a normalized collision energy of 30% and an isolation window of 2 m/z.

The mass spectrometry proteomics data have been deposited to the jPOST repository [26] with the dataset identifier JPST004803/JPST004804and the ProteomeXchange identifier PXD081727/PXD081736.

#### 2.4.4. Proteomic and Phosphoproteomic Data Processing

Peptide and protein identification were performed using MaxQuant software (version 1.6.14.0; Max Planck Institute of Biochemistry, Martinsried, Germany) [27,28]. The peak data obtained from mass spectrometry were searched against the *Homo sapiens* protein sequence database in FASTA format downloaded from the UniProt database (May 2020 release) [29]. Protein identification and peptide assignment were performed using the default search parameters in MaxQuant, including a false discovery rate (FDR) of 1% at both the peptide and protein levels, carbamidomethylation of cysteine residues as a fixed modification, and oxidation of methionine residues and protein N-terminal acetylation as variable modifications. For analysis of the phosphoproteome, phospho (STY) was added as a variable modification. Protein expression levels were quantified using the label-free quantification (LFQ) method.

The data generated by MaxQuant were further analyzed using Perseus software (version 2.1.6.0; Max Planck Institute of Biochemistry, Martinsried, Germany) [30]. Protein abundance in each sample was determined based on the label-free quantification (LFQ) intensity values. Proteins identified only by site, reverse hits, and potential contaminants were removed during the initial filtering step. Subsequently, LFQ intensity values were log2-transformed to approximate a normal distribution. Missing values were imputed from a normal distribution using a downshift of 1.8 and a width of 0.3 standard deviations. Samples were then assigned to two groups: the FIR-irradiated group and the non-irradiated control group.

Differences in protein abundance between the two groups were assessed using a two-sample Student’s *t*-test and visualized using volcano plots. Heatmaps were subsequently generated for significantly altered proteins following z-score normalization. For phosphoproteomic analysis, the Phospho (STY) Sites output generated by MaxQuant was used. Phosphorylation sites with a localization probability greater than 0.75 were retained for further analysis. After log2 transformation and expansion of the site table, the samples were assigned to the non-irradiated control and FIR-irradiated groups. Only phosphosites detected in all samples were retained for statistical analysis. Differences in phosphosite abundance between the two groups were evaluated using a two-sample Student’s *t*-test, followed by z-score normalization and heatmap generation.

Differentially expressed proteins and differentially phosphorylated sites were defined as those with an absolute log2 fold change of ≥0.585 (corresponding to a 1.5-fold change) and a *p*-value of <0.05.

### 2.5. Pathway Enrichment and Protein–Protein Interaction Network Analyses

Pathway enrichment analyses were performed using Metascape (version 3.5.20260701; https://metascape.org; accessed on 30 June 2026) [20]. Gene and protein identifiers were converted to official human gene symbols, and *Homo sapiens* was selected as both the input and analysis species. Upregulated and downregulated genes identified in human femoral artery endothelial cells following far-infrared irradiation were submitted as separate gene lists. Differentially expressed proteins identified by proteomic analysis were analyzed using the corresponding official gene symbols. Pathway enrichment analysis was performed using Reactome Gene Sets [21]. Multiple Gene List Analysis was performed to compare enriched pathways among the gene and protein datasets. Enriched pathways were considered statistically significant using the default Metascape criteria of a minimum overlap of three genes, an enrichment factor greater than 1.5, and a cumulative hypergeometric *p*-value of <0.01. Multiple-testing correction was performed using the Benjamini–Hochberg procedure [24]. Protein–protein interaction (PPI) enrichment analysis was performed using the integrated databases in Metascape, including BioGrid, InWeb_IM, and OmniPath [31,32,33]. The resulting interaction networks were visualized using the network visualization tools implemented in Metascape. Densely connected network components were identified using the Molecular Complex Detection (MCODE) algorithm, and representative biological functions were assigned to each cluster based on the most significantly enriched pathway terms [34]. Hub proteins and highly interconnected molecular modules were identified from the resulting PPI networks. For time-course phosphoproteomic analysis, significantly regulated phosphoproteins identified at 6, 12, and 24 h after FIR irradiation were analyzed separately using the Multiple Gene List Analysis module in Metascape. Pathway enrichment analysis was performed using Reactome Gene Sets, and enriched pathways were visualized as clustered heatmaps based on the −log10(*p*-value) [21]. Protein–protein interaction enrichment analysis was subsequently performed using the significantly regulated phosphoproteins identified at each time point. The resulting interaction networks were visualized using the network visualization tools implemented in Metascape. Densely connected network modules were identified using the Molecular Complex Detection (MCODE) algorithm, and network nodes were colored according to MCODE cluster membership or the time point at which phosphorylation changes were observed [34].

## 3. Results

### 3.1. Transcriptional Response of HFAECs to FIR Irradiation

**Figure 1:**
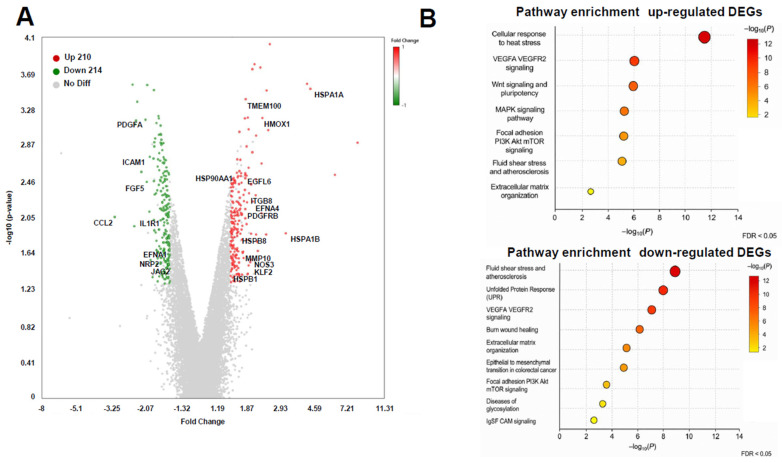
**DNA microarray analysis of HFAECs after FIR irradiation.** Transcriptional profiling using DNA microarray analysis was performed to evaluate gene expression changes in human femoral artery endothelial cells (HFAECs) after far-infrared radiation (FIR) irradiation. Among 16,081 analyzed genes, 210 genes were upregulated and 214 genes were downregulated after FIR irradiation. (**A**) The volcano plot shows the distribution of differentially expressed genes between control and FIR-irradiated HFAECs, including representative FIR-responsive genes such as EFNA4, HSPA1A, HSPA1B, HMOX1, NOS3, KLF2, TMEM100, EGFL6, HSP90AA1, PDGFRB, HSPB1, HSPB8, ITGB8, MMP10, PDGFA, IL1R1, FGF5, NRP2, JAG2, EFNA1, CCL2, and ICAM1. Red and green dots indicate significantly upregulated and downregulated genes, respectively, whereas gray dots represent genes that were not significantly changed. (**B**) Pathway enrichment analyses of upregulated and downregulated differentially expressed genes are shown as bubble plots. Bubble color indicates the significance level (−log10 (adjusted *p* value)). Abbreviations: differentially expressed genes (DEGs); far-infrared radiation (FIR); human femoral artery endothelial cells (HFAECs).

Representative phase-contrast images showed no obvious morphological alterations or cell detachment in HFAECs following FIR irradiation at any of the examined time points (Figure S1). To characterize the transcriptional response of human femoral artery endothelial cells (HFAECs) to far-infrared radiation (FIR), DNA microarray analysis was performed. Among 16,081 analyzed genes, 210 genes were upregulated and 214 genes were downregulated after FIR irradiation (Figure 1). The volcano plot and pathway enrichment analyses demonstrated that FIR irradiation induced a discrete transcriptional response in HFAECs. The differentially expressed genes included several endothelial homeostasis- and cytoprotection-related molecules, such as KLF2, NOS3, HMOX1, heat shock protein family A member 1A (HSPA1A), heat shock protein family A member 1B (HSPA1B), heat shock protein 90 alpha family class A member 1 (HSP90AA1), heat shock protein family B member 1 (HSPB1), and heat shock protein family B member 8 (HSPB8) (Figure 1). These genes are associated with endothelial protection, nitric oxide-related vascular regulation, oxidative-stress control, and heat shock responses. In addition, vascular remodeling-related genes, including ephrin A4 (EFNA4), transmembrane protein 100 (TMEM100), epidermal growth factor-like protein 6 (EGFL6), platelet-derived growth factor receptor beta (PDGFRB), integrin subunit beta 8 (ITGB8), matrix metalloproteinase 10 (MMP10), fibroblast growth factor 5 (FGF5), C-C motif chemokine ligand 2 (CCL2), and intercellular adhesion molecule 1 (ICAM1), were detected among the differentially expressed genes. Genes related to canonical angiogenic signaling and vascular patterning, including platelet-derived growth factor subunit A (PDGFA), neuropilin 2 (NRP2), jagged canonical Notch ligand 2 (JAG2), and ephrin A1 (EFNA1), were also altered (Figure 1). Thus, the transcriptomic profile indicated that FIR irradiation modulated genes related to endothelial protection, inflammatory or stress responses, extracellular matrix remodeling, and vascular signaling in HFAECs.

### 3.2. Early Proteomic Response Immediately After FIR Irradiation

**Figure 2:**
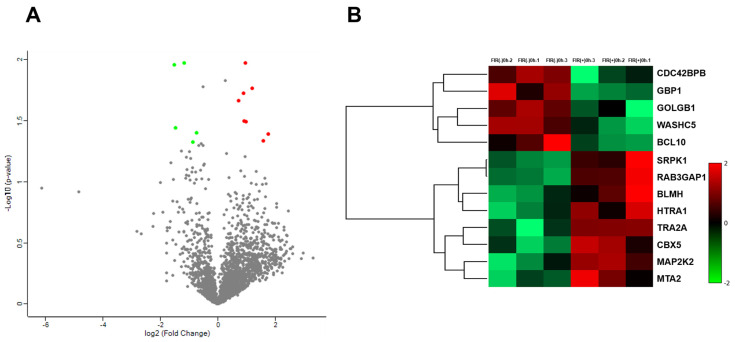
**Global proteomic response of HFAECs immediately after FIR irradiation.** Global proteomic analysis was performed immediately after FIR irradiation (0 h) to identify early changes in protein abundance in HFAECs. (**A**) The volcano plot shows the distribution of differentially abundant proteins between control and FIR-irradiated HFAECs. Red and green dots indicate significantly upregulated and downregulated proteins, respectively. (**B**) The hierarchical clustering heat map illustrates the relative abundance patterns of the differentially abundant proteins across samples. A total of 8 proteins were upregulated and 5 proteins were downregulated immediately after FIR irradiation. The differentially abundant proteins included CDC42BPB, GBP1, GOLGB1, WASHC5, BCL10, SRPK1, RAB3GAP1, BLMH, HTRA1, TRA2A, CBX5, MAP2K2, and MTA2. Among these proteins, MAP2K2, SRPK1, HTRA1, and MTA2 are associated with MEK/ERK signaling, splicing regulation, extracellular matrix remodeling, and transcriptional and chromatin regulation, respectively. Abbreviations: far-infrared radiation (FIR); human femoral artery endothelial cells (HFAECs).

Global proteomic analysis immediately after FIR irradiation identified 8 upregulated and 5 downregulated proteins (Figure 2). The differentially abundant proteins included CDC42BPB, GBP1, GOLGB1, WASHC5, BCL10, SRSF protein kinase 1 (SRPK1), RAB3GAP1, BLMH, HtrA serine peptidase 1 (HTRA1), TRA2A, CBX5, mitogen-activated protein kinase kinase 2 (MAP2K2), and metastasis-associated protein 2 (MTA2). Among these proteins, MAP2K2 was notable because of its association with MEK/ERK signaling, a pathway involved in endothelial activation, proliferation, and migration. SRPK1, which is involved in splicing regulation, HTRA1, which is associated with extracellular matrix remodeling, and MTA2, which is related to transcriptional and chromatin regulation, were also identified at this early time point (Figure 2). These findings indicate that FIR irradiation induced an immediate proteomic response involving signal priming, post-transcriptional regulation, extracellular matrix-related responses, and transcriptional regulation.

### 3.3. Proteomic Response at 6 h After FIR Irradiation

**Figure 3:**
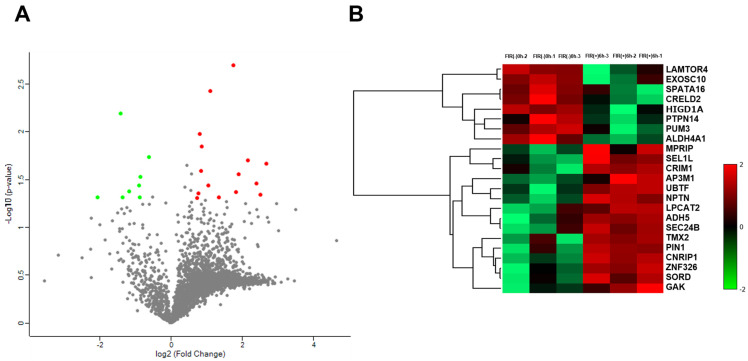
**Global proteomic response of HFAECs at 6 h after FIR irradiation.** Global proteomic analysis was performed 6 h after FIR irradiation. After filtering the protein dataset from 3656 to 2524 proteins, 15 upregulated and 8 downregulated proteins were identified in FIR-irradiated HFAECs compared with control cells. (**A**) The volcano plot shows the distribution of differentially abundant proteins between control and FIR-irradiated HFAECs. Red and green dots indicate significantly upregulated and downregulated proteins, respectively. (**B**) The hierarchical clustering heat map illustrates the relative abundance patterns of the differentially abundant proteins across samples. Representative proteins included LAMTOR4, EXOSC10, SPATA16, CRELD2, ALDH4A1, MPRIP, LPCAT2, ADH5, PTPN14, NPTN, SEL1L, PUM3, CRIM1, AP3M1, UBTF, SEC24B, TMX2, PIN1, CNRIP1, ZNF326, SORD, GAK, and HIGD1A. CRIM1, PIN1, MPRIP, ADH5, and LPCAT2 were highlighted as candidate molecules associated with vascular maturation, multipathway signal regulation, cytoskeletal remodeling, nitric oxide-related metabolism, and membrane lipid remodeling, respectively. Abbreviations: far-infrared radiation (FIR); human femoral artery endothelial cells (HFAECs).

At 6 h after FIR irradiation, global proteomic analysis was performed after filtering the protein dataset from 3656 to 2524 proteins. This analysis identified 15 upregulated and 8 downregulated proteins (Figure 3). The differentially abundant proteins included LAMTOR4, EXOSC10, CRELD2, ALDH4A1, myosin phosphatase Rho-interacting protein (MPRIP), lysophosphatidylcholine acyltransferase 2 (LPCAT2), alcohol dehydrogenase 5 (ADH5), PTPN14, NPTN, cysteine-rich transmembrane bone morphogenetic protein regulator 1 (CRIM1), AP3M1, SEC24B, peptidyl-prolyl cis-trans isomerase NIMA-interacting 1 (PIN1), and GAK. Several of these proteins were related to vascular maturation, intracellular trafficking, cytoskeletal regulation, and endothelial functional control. CRIM1 was identified as a vascular formation- and vascular maturation-related molecule. PIN1 was associated with regulation of multiple signaling pathways, including MEK/ERK, β-catenin, Notch, hypoxia-inducible factor 1α (HIF-1α), and VEGF-related signaling. MPRIP was related to Rho/Rho-associated coiled-coil-containing protein kinase (ROCK)-mediated cytoskeletal remodeling, whereas ADH5 was associated with nitric oxide-related metabolism and oxidative-stress regulation. LPCAT2 was related to lipid signaling and membrane remodeling (Figure 3). These results indicate that, by 6 h after FIR irradiation, HFAECs exhibited a proteomic response involving vascular maturation-related signaling, cytoskeletal remodeling, nitric oxide-related regulation, intracellular trafficking, and membrane-associated responses.

### 3.4. Proteomic Response at 12 h After FIR Irradiation

**Figure 4:**
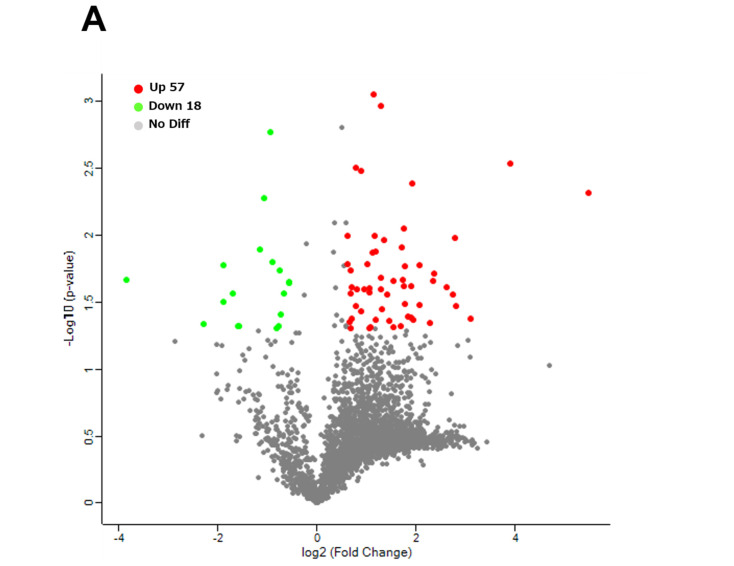

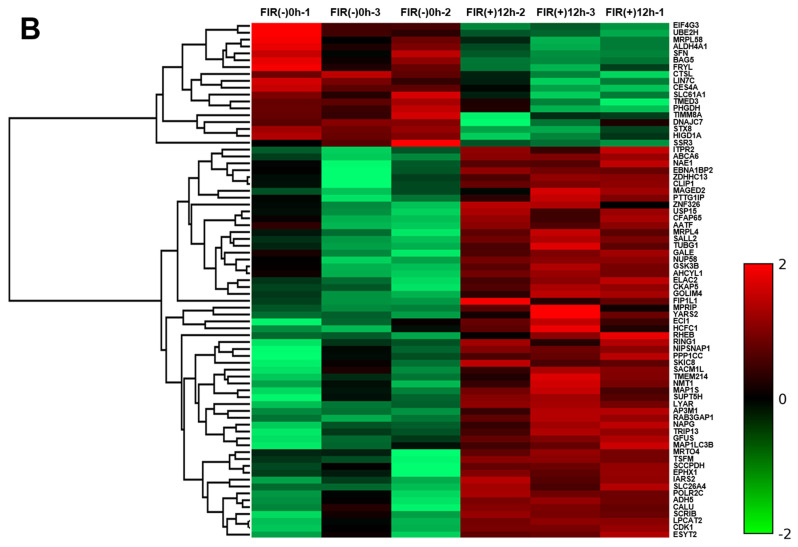
**Global proteomic response of HFAECs at 12 h after FIR irradiation.** Global proteomic analysis was performed 12 h after FIR irradiation. After filtering the protein dataset from 3656 to 2648 proteins, 57 upregulated and 18 downregulated proteins were identified in FIR-irradiated HFAECs compared with control cells. (**A**) The volcano plot shows the distribution of differentially abundant proteins between control and FIR-irradiated HFAECs. Red and green dots indicate significantly upregulated and downregulated proteins, respectively. (**B**) The hierarchical clustering heat map illustrates the relative abundance patterns of the differentially abundant proteins across samples. Representative proteins included EIF4G3, UBE2H, SFN, CKAP5, RHEB, GSK3B, DNAJC7, ITPR2, USP15, MPRIP, MAP1LC3B, ADH5, LPCAT2, SCRIB, and CDK1. Functional annotation of the differentially abundant proteins indicated modules related to cell polarity and cytoskeletal remodeling, Wnt/β-catenin and TGF-β/BMP-associated signaling, Ca^2+^/NO-related endothelial regulation, and mTOR/autophagy-associated cellular adaptation. Abbreviations: far-infrared radiation (FIR); human femoral artery endothelial cells (HFAECs).

At 12 h after FIR irradiation, the protein dataset was filtered from 3656 to 2648 proteins, and 57 upregulated and 18 downregulated proteins were identified (Figure 4). This represented a broader proteomic alteration than that observed at 6 h. The differentially abundant proteins included molecules related to cell polarity, cytoskeletal remodeling, Wnt/TGF-β-related signaling, Ca^2+^/NO signaling, and adaptive cellular responses. Scribble planar cell polarity protein (SCRIB) and MPRIP were associated with cell polarity, actin cytoskeletal remodeling, and endothelial migration. Glycogen synthase kinase 3 beta (GSK3B) and ubiquitin-specific peptidase 15 (USP15) were related to Wnt/β-catenin and TGF-β/BMP-associated signaling. Inositol 1,4,5-trisphosphate receptor type 2 (ITPR2) and ADH5 were associated with Ca^2+^ signaling and nitric oxide-related endothelial regulation. Ras homolog, mechanistic target of rapamycin complex 1 binding (RHEB) and microtubule-associated protein 1 light chain 3 beta (MAP1LC3B) were related to mechanistic target of rapamycin (mTOR) signaling and autophagy-associated adaptation (Figure 4). These findings indicate that FIR irradiation induced a coordinated proteomic response at 12 h, involving cell polarity, cytoskeletal organization, noncanonical vascular signaling pathways, Ca^2+^/NO-related endothelial regulation, and adaptive survival-related processes.

### 3.5. Proteomic Response at 24 h After FIR Irradiation

**Figure 5:**
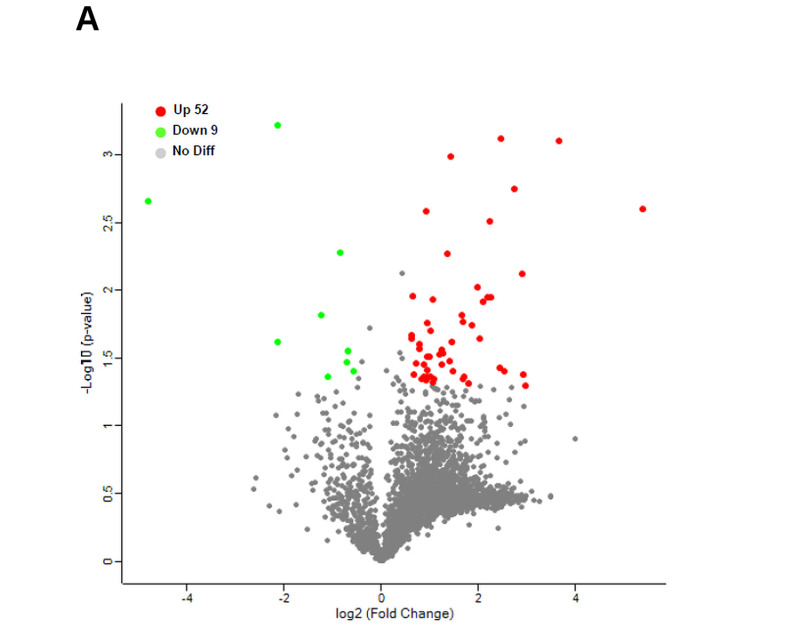

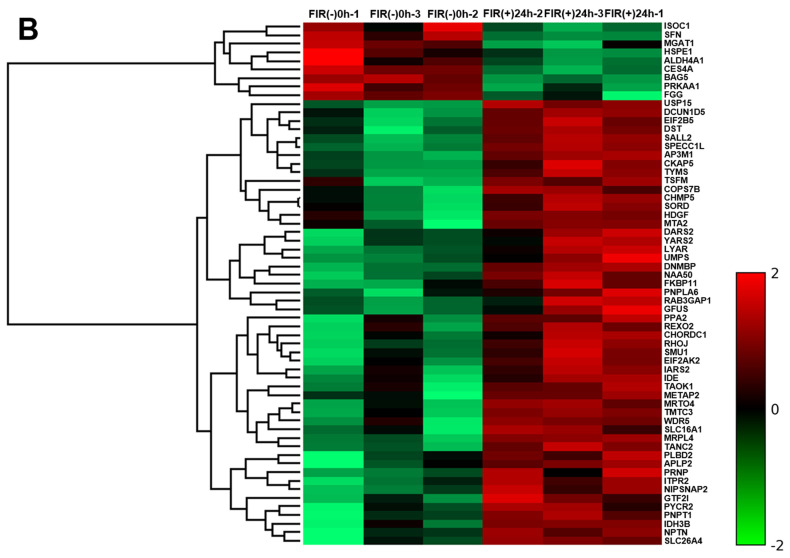
**Global proteomic response of HFAECs at 24 h after FIR irradiation** Global proteomic analysis was performed 24 h after FIR irradiation. After filtering the protein dataset from 3537 to 2639 proteins, 52 upregulated and 9 downregulated proteins were identified in FIR-irradiated HFAECs compared with control cells. (**A**) The volcano plot shows the distribution of differentially abundant proteins between control and FIR-irradiated HFAECs. Red and green dots indicate significantly upregulated and downregulated proteins, respectively. (**B**) The hierarchical clustering heat map illustrates the relative abundance patterns of the differentially abundant proteins across samples. Representative proteins included SFN, USP15, FKBP11, TAOK1, PRKAA1, HSPE1, HDGF, MTA2, DNMBP, RHOJ, ITPR2, SLC16A1, METAP2, EIF2AK2, CHORDC1, and WDR5. The 24 h proteomic response included proteins associated with endothelial migration and cytoskeletal remodeling, VEGF-independent or VEGF-noncentral angiogenesis-supportive signaling, Ca^2+^/NO-related endothelial regulation, and metabolic or stress-adaptive responses. Abbreviations: far-infrared radiation (FIR); human femoral artery endothelial cells (HFAECs).

At 24 h after FIR irradiation, global proteomic analysis was performed after filtering the protein dataset from 3537 to 2639 proteins. A total of 52 upregulated and 9 downregulated proteins were identified (Figure 5). The differentially abundant proteins included SFN, USP15, TAOK1, PRKAA1, hepatoma-derived growth factor (HDGF), MTA2, dynamin-binding protein (DNMBP), Ras homolog family member J (RHOJ), ITPR2, solute carrier family 16 member 1 (SLC16A1), methionyl aminopeptidase 2 (METAP2), eukaryotic translation initiation factor 2 alpha kinase 2 (EIF2AK2), cysteine and histidine-rich domain-containing 1 (CHORDC1), FKBP prolyl isomerase 11 (FKBP11), and WD repeat domain 5 (WDR5). Proteins related to endothelial migration and cytoskeletal remodeling included RHOJ, DNMBP, cytoskeleton-associated protein 5 (CKAP5), and dystonin (DST). HDGF, USP15, METAP2, and MTA2 were associated with VEGF-independent or VEGF-noncentral angiogenesis-supportive signaling. ITPR2 was related to Ca^2+^-dependent endothelial regulation, potentially in association with nitric oxide-related pathways. SLC16A1, EIF2AK2, CHORDC1, and FKBP11 were associated with metabolic adaptation, stress responses, and proteostasis (Figure 5). Thus, the 24 h proteomic response was characterized by coordinated changes in endothelial migration, cytoskeletal remodeling, VEGF-noncentral vascular signaling, Ca^2+^/NO-related endothelial regulation, and metabolic or stress-adaptive processes.

### 3.6. Phosphoproteomic Response at 6 h After FIR Irradiation

**Figure 6:**
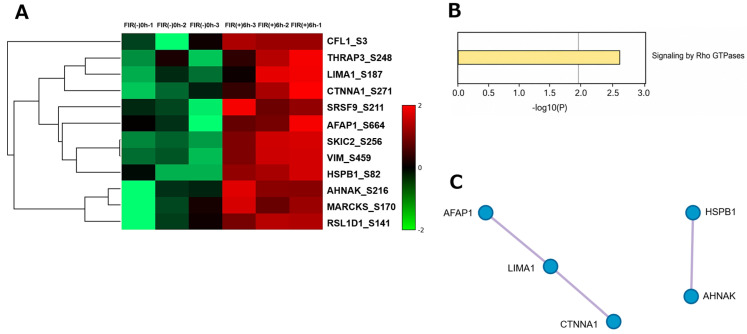
**Global phosphoproteomic response of HFAECs at 6 h after FIR irradiation.** Global phosphoproteomic analysis was performed 6 h after FIR irradiation. (**A**) The hierarchical clustering heat map illustrates the relative phosphorylation patterns of the differentially phosphorylated proteins across samples. (**B**) Reactome pathway enrichment analysis of the differentially phosphorylated proteins is shown. (**C**) Protein–protein interaction (PPI) network analysis of the differentially phosphorylated proteins is shown. A total of 12 differentially phosphorylated proteins were identified in FIR-irradiated HFAECs compared with control cells, including CFL1, THRAP3, LIMA1, CTNNA1, SRSF9, AFAP1, SKIC2, VIM, HSPB1, AHNAK, MARCKS, and RSL1D1. PPI network analysis identified a small interaction network involving AFAP1, LIMA1, CTNNA1, HSPB1, and AHNAK. Functional enrichment analysis indicated that these phosphorylation changes were primarily associated with actin cytoskeletal remodeling, endothelial cell adhesion, membrane–cytoskeletal organization, and stress-adaptive cytoskeletal regulation. Abbreviations: far-infrared radiation (FIR); human femoral artery endothelial cells (HFAECs); protein–protein interaction (PPI).

Global phosphoproteomic analysis at 6 h after FIR irradiation identified 12 phosphorylation-related changes (Figure 6). Reactome pathway enrichment, hierarchical clustering, and protein–protein interaction (PPI) network analysis showed that these changes were mainly associated with cytoskeletal remodeling and cell adhesion. The differentially phosphorylated proteins included cofilin 1 (CFL1), THRAP3, LIM domain and actin-binding protein 1 (LIMA1), catenin alpha 1 (CTNNA1), SRSF9, actin filament-associated protein 1 (AFAP1), SKIC2, vimentin (VIM), HSPB1, AHNAK nucleoprotein (AHNAK), myristoylated alanine-rich protein kinase C substrate (MARCKS), and RSL1D1. Among these molecules, CFL1, LIMA1, VIM, AFAP1, and MARCKS were related to actin dynamics and cytoskeletal organization. CTNNA1 and AHNAK were associated with cell–cell adhesion and membrane cytoskeletal organization, whereas HSPB1 was related to stress responses and actin regulation (Figure 6). These findings indicate that FIR irradiation induced early phosphorylation changes related to actin cytoskeletal remodeling, endothelial cell adhesion, membrane organization, and stress-adaptive cytoskeletal regulation.

### 3.7. Phosphoproteomic Response at 12 h After FIR Irradiation

**Figure 7:**
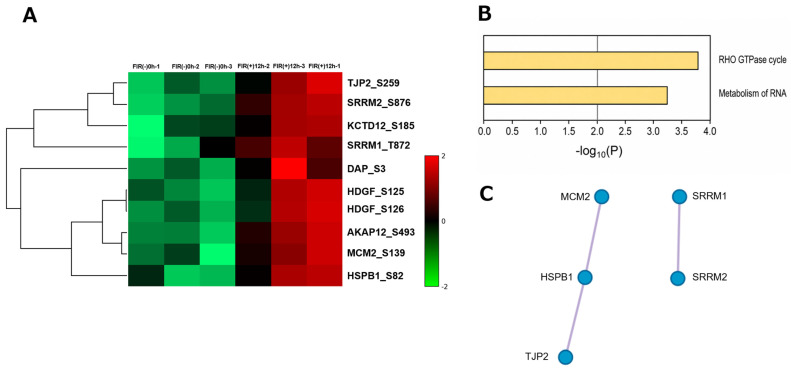
**Global phosphoproteomic response of HFAECs at 12 h after FIR irradiation.** Global phosphoproteomic analysis was performed 12 h after FIR irradiation. (**A**) The hierarchical clustering heat map illustrates the relative phosphorylation patterns of the differentially phosphorylated proteins across samples. (**B**) Reactome pathway enrichment analysis of the differentially phosphorylated proteins. (**C**) Protein–protein interaction (PPI) network analysis of the differentially phosphorylated proteins. A total of 10 differentially phosphorylated sites corresponding to 9 proteins were identified in FIR-irradiated HFAECs compared with control cells. The differentially phosphorylated proteins included TJP2, SRRM2, KCTD12, SRRM1, DAP, HDGF, AKAP12, MCM2, and HSPB1. PPI network analysis highlighted MCM2, HSPB1, TJP2, SRRM1, and SRRM2. The 12 h phosphoproteomic response was characterized by proteins related to junction remodeling, endothelial structural stabilization, cytoskeletal regulation, stress adaptation, and HDGF-associated endothelial remodeling. Abbreviations: far-infrared radiation (FIR); human femoral artery endothelial cells (HFAECs); protein–protein interaction (PPI); tight junction protein 2 (TJP2).

At 12 h after FIR irradiation, global phosphoproteomic analysis identified 10 phosphorylation-related changes (Figure 7). The differentially phosphorylated proteins included tight junction protein 2 (TJP2), SRRM2, KCTD12, SRRM1, DAP, HDGF, A-kinase anchoring protein 12 (AKAP12), MCM2, and HSPB1. HDGF was identified as a phosphorylation-regulated molecule associated with endothelial proliferation and migration. HSPB1 was related to stress responses and actin cytoskeletal regulation. AKAP12 was associated with protein kinase A (PKA)/Src-related signaling, cell adhesion, and cytoskeletal control. TJP2, also known as zonula occludens-2 (ZO-2), is a tight junction-related molecule involved in endothelial junctional organization (Figure 7). These results indicate that the 12 h phosphoproteomic response was characterized by changes related to junction remodeling, endothelial structural stabilization, cytoskeletal regulation, stress adaptation, and HDGF-associated endothelial remodeling.

### 3.8. Phosphoproteomic Response at 24 h After FIR Irradiation

**Figure 8:**
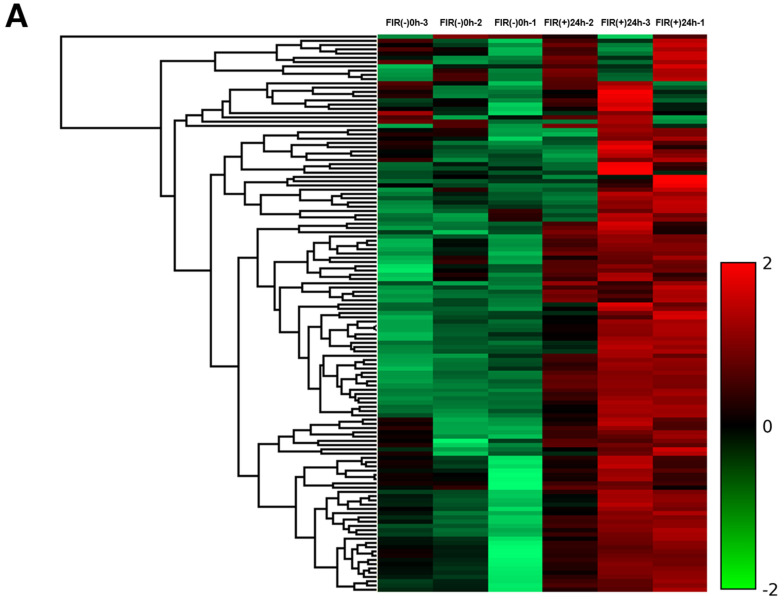

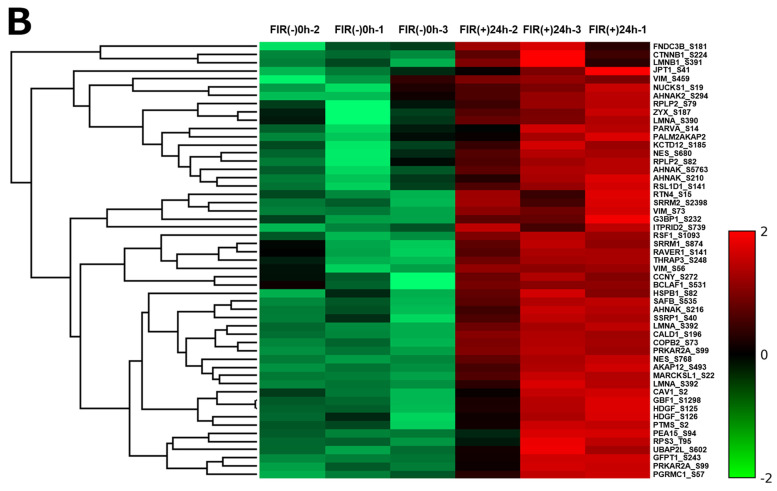

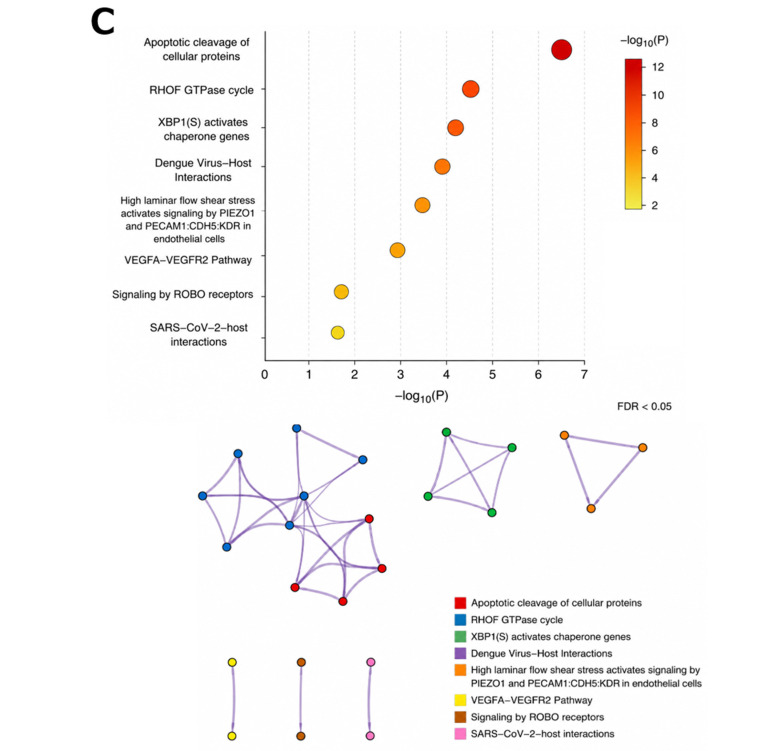
**Global phosphoproteomic response of HFAECs at 24 h after FIR irradiation.** Global phosphoproteomic analysis was performed 24 h after FIR irradiation. (**A**) The hierarchical clustering heat map illustrates the phosphorylation patterns of all identified phosphorylation sites across samples. (**B**) The hierarchical clustering heat map illustrates the relative phosphorylation patterns of the differentially phosphorylated sites across samples. (**C**) Reactome pathway enrichment analysis and protein–protein interaction (PPI) network analysis of the differentially phosphorylated proteins. A total of 131 phosphorylation sites were identified in HFAECs (**A**), of which 53 were differentially phosphorylated following FIR irradiation compared with control cells (**B**). Representative differentially phosphorylated proteins included LMNB1, FNDC3B, VIM, RPLP2, NUCKS1, CTNNB1, PARVA, AHNAK, HDGF, CAV1, ZYX, NES, SAFB, AHNAK2, LIMA1, LMNA, SRRM1, SRRM2, G3BP1, HSPB1, CALD1, MARCKSL1, AKAP12, RPS3, and PEA15. Reactome pathway enrichment analysis identified significant enrichment of pathways related to apoptotic cleavage of cellular proteins, RHOF GTPase cycle, XBP1(S)-mediated chaperone activation, VEGFA–VEGFR2 signaling, and high laminar flow shear stress signaling. PPI network analysis identified several interconnected protein modules associated with these pathways. Integrated pathway and network analyses indicated that the 24-h phosphoproteomic response was associated with mechanosensing, Wnt/β-catenin-related adhesion signaling, focal adhesion remodeling, cytoskeletal reorganization, and VEGF-independent endothelial remodeling. Abbreviations: far-infrared radiation (FIR); human femoral artery endothelial cells (HFAECs); protein–protein interaction (PPI).

At 24 h after FIR irradiation, global phosphoproteomic analysis identified a total of 131 phosphorylation sites in HFAECs (Figure 8A), of which 53 were differentially phosphorylated following FIR irradiation compared with control cells (Figure 8B). The differentially phosphorylated proteins included lamin B1 (LMNB1), FNDC3B, VIM, ribosomal protein lateral stalk subunit P2 (RPLP2), NUCKS1, CTNNB1, KCTD12, parvin alpha (PARVA), AHNAK, HDGF, CAV1, zyxin (ZYX), nestin (NES), SAFB, AHNAK2, LIMA1, lamin A/C (LMNA), SRRM1, SRRM2, G3BP stress granule assembly factor 1 (G3BP1), HSPB1, caldesmon 1 (CALD1), MARCKS-like protein 1 (MARCKSL1), AKAP12, ribosomal protein S3 (RPS3), and proliferation and apoptosis adaptor protein 15 (PEA15). Several of these molecules were associated with vascular structural maturation, mechanotransduction, and focal adhesion remodeling. HDGF was related to VEGF-independent endothelial remodeling. CTNNB1 was associated with Wnt/β-catenin signaling and cell–cell adhesion. CAV1 was related to caveolae formation, membrane signaling, and mechanosensing. PARVA, ZYX, VIM, LIMA1, CALD1, and MARCKSL1 were associated with focal adhesion and actin cytoskeletal remodeling. NES and AKAP12 were associated with structural stabilization and cytoskeletal or adhesion-related regulation (Figure 8B). These findings indicate that FIR irradiation induced a late phosphoproteomic response characterized by coordinated regulation of mechanosensing, Wnt/β-catenin-related adhesion signaling, focal adhesion maturation, cytoskeletal remodeling, and VEGF-independent endothelial remodeling.

### 3.9. PPI and Molecular Complex Detection (MCODE) Analysis of 24 h Phosphoproteomic Response

**Figure 9:**
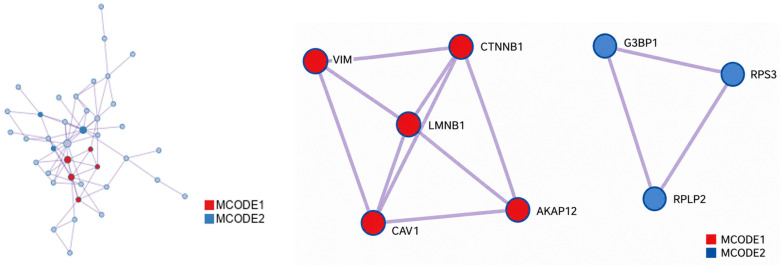
**PPI network and MCODE cluster analysis of the 24 h phosphoproteomic response to FIR irradiation in HFAECs.** Protein–protein interaction (PPI) network and Molecular Complex Detection (MCODE) analyses were performed using the differentially phosphorylated proteins identified 24 h after FIR irradiation. Two major MCODE clusters were identified. MCODE1 consisted of 5 nodes and 9 edges, with CAV1, CTNNB1, and LMNB1 serving as hub proteins, and was primarily associated with Signaling by Rho GTPases. MCODE2 consisted of 3 nodes and 3 edges and included G3BP1, RPLP2, and RPS3, representing a protein module associated with stress responses and RNA-related processes. These findings suggest that the late phosphoproteomic response to FIR irradiation involved a Rho GTPase-associated signaling network centered on CAV1, CTNNB1, and LMNB1, together with a distinct protein module associated with stress responses and RNA-related processes. Abbreviations: far-infrared radiation (FIR); human femoral artery endothelial cells (HFAECs); Molecular Complex Detection (MCODE); protein–protein interaction (PPI).

PPI network and MCODE cluster analysis of the 24 h phosphoproteomic response identified two major clusters (Figure 9). MCODE1 consisted of 5 nodes and 9 edges, with CAV1, CTNNB1, and LMNB1 identified as hub proteins. The representative pathway for this cluster was Signaling by Rho GTPases. MCODE2 consisted of 3 nodes and 3 edges, included G3BP1, RPLP2, and RPS3, and was associated with cellular responses to stress and RNA-related responses (Figure 9). The identification of CAV1, CTNNB1, and LMNB1 as hub proteins suggests that the 24 h phosphoproteomic response involved a network related to membrane mechanosensing, cell adhesion, cytoskeletal organization, and nuclear structural regulation. The second cluster suggests that stress-related and RNA-related responses were also present in parallel with the structural remodeling network (Figure 9).

### 3.10. Time-Dependent Rho GTPase-Related Phosphorylation Network After FIR Irradiation

**Figure 10:**
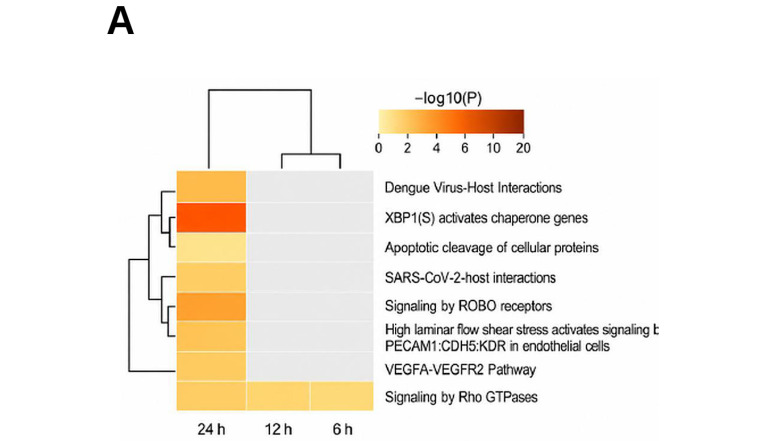

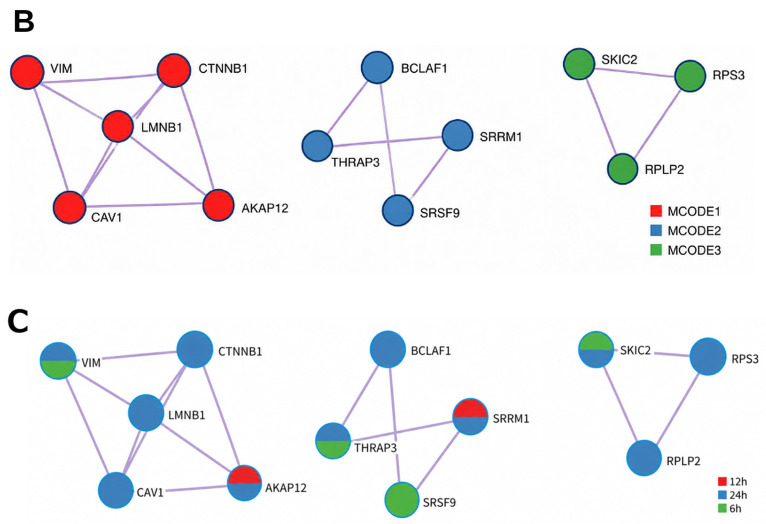
**Time-dependent activation of the Rho GTPase-related phosphorylation network after FIR irradiation in HFAECs.** Integrated phosphoproteomic analysis was performed to evaluate the temporal activation of phosphorylation networks following FIR irradiation. (**A**) Reactome pathway enrichment analysis summarizing the major pathways enriched at 6, 12, and 24 h after FIR irradiation. (**B**) MCODE cluster analysis of the integrated PPI network generated from differentially phosphorylated proteins identified at 6, 12, and 24 h after FIR irradiation. (**C**) Integrated time-course phosphorylation network illustrating the temporal distribution of the major MCODE clusters across 6, 12, and 24 h. Reactome pathway enrichment and MCODE cluster analyses demonstrated sequential activation of a Rho GTPase-associated phosphorylation network in HFAECs. At 6 h, phosphorylation changes were primarily associated with cytoskeletal remodeling and cell adhesion. At 12 h, the response shifted toward junction remodeling and endothelial structural stabilization. At 24 h, phosphorylation changes were associated with CAV1/CTNNB1-centered mechanotransduction, focal adhesion remodeling, cytoskeletal maturation, RNA processing, and translation-related processes. These findings indicate that FIR irradiation induced a time-dependent phosphoproteomic program progressing from early cytoskeletal and adhesion-related responses to Rho GTPase-associated mechanotransduction and structural remodeling. Abbreviations: far-infrared radiation (FIR); human femoral artery endothelial cells (HFAECs); Molecular Complex Detection (MCODE).

Integrated analysis of Reactome enrichment, time-course phosphorylation networks, and MCODE networks demonstrated time-dependent activation of a Rho GTPase-related phosphorylation network after FIR irradiation (Figure 10). This network was associated with cytoskeletal remodeling, RNA processing, and translation-related processes. At 6 h, phosphorylation changes were mainly related to actin dynamics, cytoskeletal remodeling, and cell adhesion, as represented by CFL1, LIMA1, VIM, AFAP1, MARCKS, CTNNA1, AHNAK, and HSPB1 (Figure 6). At 12 h, the phosphorylation response shifted toward junction remodeling and endothelial structural stabilization, involving HDGF, AKAP12, TJP2, and HSPB1 (Figure 7). At 24 h, phosphorylation changes involving CAV1, CTNNB1, PARVA, ZYX, VIM, LIMA1, CALD1, HDGF, and AKAP12 indicated structural maturation, mechanosensing, focal adhesion remodeling, and cytoskeletal reorganization (Figure 8 and Figure 9). Collectively, the phosphoproteomic data indicate a sequential phosphorylation program in FIR-irradiated HFAECs, beginning with cytoskeletal and adhesion-related responses, progressing through junction remodeling, and culminating in Rho GTPase-related mechanotransduction and structural maturation.

## 4. Discussion

This study characterized the transcriptional, proteomic, and phosphoproteomic responses of human femoral artery endothelial cells (HFAECs) to far-infrared radiation (FIR). The integrated design therefore comprised three complementary omics platforms rather than a single analytical representation and enabled time-resolved assessment of molecular changes after FIR exposure. The principal finding was a temporally organized molecular response involving endothelial-protective and nitric oxide-related signatures, early MEK/ERK-related signal priming, cytoskeletal and junctional remodeling, VEGF-noncentral vascular signaling, and late Rho GTPase-related structural adaptation. These findings provide a mechanistic and hypothesis-generating framework; however, the omics data alone do not establish that FIR improves endothelial function or vascular repair, which requires direct functional validation.

The DNA microarray analysis showed that FIR irradiation altered the expression of 424 genes, including 210 upregulated and 214 downregulated genes among 16,081 analyzed genes (Figure 1). The differentially expressed genes included KLF2, NOS3, HMOX1, and several heat shock protein-related genes, such as HSPA1A, HSPA1B, HSP90AA1, HSPB1, and HSPB8. This transcriptional profile suggests that the initial response of HFAECs to FIR irradiation was characterized by endothelial protection, nitric oxide-related regulation, oxidative-stress control, and proteostasis. In particular, KLF2 and NOS3 are highly relevant to arterial endothelial homeostasis, because KLF2 is a key regulator of anti-inflammatory and flow-responsive endothelial phenotypes, whereas NOS3 mediates nitric oxide production and vascular protective functions [7,18]. Therefore, the transcriptional response observed in HFAECs appears to reflect a protective arterial endothelial phenotype rather than an injury-dominant response.

In addition to cytoprotective genes, the transcriptomic response included genes related to vascular remodeling, extracellular matrix regulation, and endothelial signaling, such as EGFL6, PDGFRB, TMEM100, ITGB8, MMP10, and FGF5 [7,15]. These molecules suggest that FIR irradiation may create a molecular environment permissive for endothelial repair and vascular maturation. However, several molecules associated with canonical angiogenic signaling or vascular patterning, including PDGFA, NRP2, JAG2, and EFNA1, were also altered [7,15]. Therefore, the transcriptional response should not be interpreted as straightforward activation of classical VEGF/Notch/Ephrin-dependent sprouting angiogenesis. Rather, FIR irradiation may preferentially induce an endothelial-protective and vascular repair-associated transcriptional state in HFAECs.

Immediately after FIR irradiation, proteomic analysis identified 8 upregulated and 5 downregulated proteins (Figure 2). Among these, MAP2K2 was notable because it is a component of the MEK/ERK pathway, which participates in endothelial activation, migration, proliferation, and remodeling [5,7]. The early alteration of MAP2K2 suggests that FIR irradiation may rapidly prime MEK/ERK-related signaling in HFAECs. This may represent an early signaling state that prepares cells for subsequent structural and functional remodeling. In addition, SRPK1, HTRA1, and MTA2 suggest the involvement of post-transcriptional regulation, extracellular matrix remodeling, and transcriptional or chromatin regulation at the immediate phase after FIR exposure [20,21,29].

At 6 h after FIR irradiation, 15 upregulated and 8 downregulated proteins were identified after filtering the dataset from 3,656 to 2,524 proteins (Figure 3). This phase was characterized by proteins related to vascular maturation, cytoskeletal regulation, nitric oxide-related metabolism, intracellular trafficking, and membrane remodeling. CRIM1 may be particularly relevant because of its association with vascular formation and maturation [35]. PIN1 may function as a signaling integrator connecting MEK/ERK, β-catenin, Notch, HIF-1α, and VEGF-related pathways [36]. MPRIP may contribute to actin cytoskeletal remodeling through Rho/ROCK-related mechanisms, whereas ADH5 may connect the proteomic response to nitric oxide-related regulation [37,38]. These findings suggest that, by 6 h, HFAECs had progressed from early signal priming toward a more organized endothelial remodeling response.

At 12 h after FIR irradiation, the proteomic response expanded substantially, with 57 upregulated and 18 downregulated proteins identified after filtering the dataset from 3,656 to 2,648 proteins (Figure 4). This broader response suggests that HFAECs entered a more active remodeling phase. The differentially abundant proteins could be organized into several functional modules, including SCRIB/MPRIP-related cell polarity and cytoskeletal remodeling, GSK3B/USP15-related Wnt/TGF-β-associated vascular signaling, ITPR2/ADH5-related Ca^2+^/NO regulation, and RHEB/MAP1LC3B-related survival and adaptive responses [39,40,41]. These findings indicate that FIR-induced remodeling in HFAECs is unlikely to be mediated by a single pathway. Instead, the response appears to involve coordinated regulation of polarity, cytoskeletal organization, calcium signaling, nitric oxide-related endothelial function, and adaptive cellular homeostasis.

At 24 h after FIR irradiation, proteomic analysis identified 52 upregulated and 9 downregulated proteins after filtering the dataset from 3537 to 2639 proteins (Figure 5). This phase was characterized by proteins associated with endothelial migration, cytoskeletal remodeling, VEGF-noncentral vascular signaling, Ca^2+^/NO-related regulation, and metabolic or stress adaptation. RHOJ and DNMBP may contribute to endothelial migration and directional cytoskeletal regulation through Rho GTPase- and cell division cycle 42 (CDC42)-related pathways [19,42]. HDGF is particularly notable because it may support endothelial proliferation and migration through VEGF-independent mechanisms [43]. The detection of HDGF, together with USP15, METAP2, MTA2, and ITPR2, suggests that the late proteomic response may support vascular remodeling through mechanisms that are not solely dependent on classical VEGF signaling [29,40,41]. This interpretation is consistent with the transcriptomic finding that some canonical angiogenic regulators were not uniformly increased.

The phosphoproteomic analyses provided additional insight into the temporal organization of FIR-induced signaling. At 6 h, 12 phosphorylation-related changes were detected and were mainly associated with cytoskeletal remodeling and cell adhesion (Figure 6). Molecules such as CFL1, LIMA1, VIM, AFAP1, MARCKS, CTNNA1, AHNAK, and HSPB1 suggest early regulation of actin dynamics, membrane-cytoskeletal organization, cell–cell adhesion, and stress-adaptive cytoskeletal control [19,37,39]. These findings indicate that structural remodeling after FIR irradiation begins not only at the protein abundance level but also at the phosphorylation level during the early response phase.

At 12 h, 10 phosphorylation-related changes were detected, including HDGF, AKAP12, TJP2, and HSPB1 (Figure 7). This phase appears to represent a transition from early cytoskeletal remodeling to junctional reorganization and endothelial structural stabilization. TJP2, also known as ZO-2, is associated with tight junction organization, whereas AKAP12 is involved in cell adhesion and cytoskeletal regulation through PKA/Src-related signaling [44,45]. The presence of HDGF in the 12 h phosphoproteomic response is notable because HDGF was also identified in the 24 h proteomic response [43]. This suggests that HDGF may be involved across multiple phases of the FIR-induced endothelial remodeling program.

At 24 h, phosphoproteomic analysis identified 53 phosphorylation-related changes (Figure 8). This late phosphorylation response included CAV1, CTNNB1, HDGF, PARVA, ZYX, VIM, LIMA1, CALD1, NES, AKAP12, AHNAK, and MARCKSL1. These molecules are closely related to caveolae formation, mechanosensing, Wnt/β-catenin-associated adhesion signaling, focal adhesion maturation, cytoskeletal remodeling, and endothelial structural stabilization [7,19,44]. In particular, CAV1 and CTNNB1 may represent key molecules linking membrane mechanosensing, cell–cell adhesion, and cytoskeletal remodeling [7,19,46]. Therefore, the 24 h phosphoproteomic profile suggests that FIR irradiation may culminate in a structural maturation-type endothelial response.

The PPI and MCODE analysis further supported the central role of Rho GTPase-related signaling in the late FIR response. At 24 h, MCODE1 consisted of 5 nodes and 9 edges, with CAV1, CTNNB1, and LMNB1 identified as hub proteins, and the representative pathway was Signaling by Rho GTPases (Figure 9). CAV1 is associated with caveolae and membrane mechanosensing, CTNNB1 with cell adhesion and Wnt/β-catenin signaling, and LMNB1 with nuclear structure and mechanical responses [7,19,46]. Thus, this cluster suggests that the late response to FIR irradiation involves an integrated mechanotransduction network connecting the plasma membrane, adhesion machinery, cytoskeleton, and nuclear architecture. MCODE2, consisting of 3 nodes and 3 edges and including G3BP1, RPLP2, and RPS3, was associated with cellular responses to stress and RNA-related responses, suggesting that stress-adaptive and RNA-related processes may occur in parallel with structural remodeling.

The integrated time-course phosphoproteomic analysis demonstrated sequential activation of a Rho GTPase-related phosphorylation network after FIR irradiation (Figure 10). At 6 h, phosphorylation changes were mainly associated with actin dynamics and cell adhesion. At 12 h, the response shifted toward junction remodeling and endothelial structural stabilization. At 24 h, phosphorylation changes were associated with CAV1/CTNNB1-centered mechanosensing, focal adhesion remodeling, and cytoskeletal maturation. These findings suggest that Rho GTPase-related signaling may act as a central organizing network that coordinates the transition from early cytoskeletal activation to late structural maturation in FIR-irradiated HFAECs [19].

Taken together, the molecular response of HFAECs to FIR irradiation appears to follow a stepwise sequence. First, FIR irradiation induces a KLF2–NOS3–HMOX1-centered endothelial-protective transcriptional response. Second, immediate MAP2K2 alteration suggests early MEK/ERK signal priming. Third, the 6 h proteomic response indicates vascular maturation-related signaling and cytoskeletal preparation through molecules such as CRIM1, PIN1, MPRIP, ADH5, and LPCAT2. Fourth, the 12 h proteomic response indicates broader remodeling involving cell polarity, Wnt/TGF-β-related signaling, Ca^2+^/NO regulation, and adaptive survival pathways. Fifth, the 24 h proteomic and phosphoproteomic responses suggest VEGF-noncentral angiogenesis-supportive signaling and structural maturation through RHOJ, HDGF, DNMBP, CAV1, CTNNB1, PARVA, ZYX, VIM, LIMA1, and CALD1.

These results also suggest that FIR irradiation may affect HFAECs differently from more developmentally plastic endothelial cell types. HFAECs are adult arterial endothelial cells and are expected to prioritize vascular homeostasis, mechanosensing, barrier stability, and antithrombotic and anti-inflammatory regulation [7,16,18]. Therefore, the observed sequence of KLF2–NOS3–HMOX1-centered protection followed by Rho GTPase-related structural maturation is biologically plausible for arterial endothelial cells. However, direct comparison with other endothelial cell types requires parallel validation under identical experimental conditions.

The present study has several limitations. First, although three complementary omics platforms were integrated, these analyses identify molecular associations and temporal signatures rather than directly demonstrating angiogenesis, migration, barrier recovery, or vascular repair. Changes in mRNA abundance, protein abundance, or phosphorylation status do not necessarily establish functional activation of the corresponding pathways. Functional assays—including tube formation, scratch wound-healing, transwell migration, nitric oxide production, reactive oxygen species measurement, cell viability, and endothelial barrier function assays—are therefore required to determine whether the observed molecular response translates into enhanced endothelial repair capacity. Targeted perturbation experiments, including MEK/ERK or Rho GTPase inhibition and knockdown of CAV1, CTNNB1, HDGF, or RHOJ, would further clarify whether the candidate pathways identified in Figure 1, Figure 2, Figure 3, Figure 4, Figure 5, Figure 6, Figure 7, Figure 8, Figure 9 and Figure 10 are functionally required for FIR-induced endothelial remodeling.

Second, no targeted fluorescence imaging or orthogonal biochemical validation was performed for selected molecules. Immunofluorescence or confocal microscopy would be valuable for confirming the abundance and, where relevant, subcellular localization of representative factors identified across the datasets, including KLF2, NOS3, HMOX1, CAV1, and CTNNB1. Quantitative PCR and immunoblotting should also be combined with functional assays to validate the principal molecular findings.

Third, only one FIR exposure condition was evaluated. Accordingly, the present findings cannot define an optimal exposure parameter or be generalized to other irradiation durations, emitter-to-sample distances, irradiances, or cumulative energy densities. Future studies should incorporate systematic single-factor dose–response experiments, real-time monitoring of culture-medium temperature, and an appropriate thermal-matched control to distinguish FIR-associated effects from nonspecific heating.

## 5. Conclusions

This integrated multi-omics study showed that FIR irradiation elicited a temporally organized molecular response in HFAECs. The early transcriptional response comprised genes associated with endothelial protection, nitric oxide regulation, oxidative-stress responses, and heat-shock adaptation, followed by immediate MEK/ERK-related signal priming. Subsequent proteomic and phosphoproteomic changes were associated with cytoskeletal remodeling, cell polarity, junctional organization, calcium/nitric oxide regulation, focal adhesion maturation, and mechanotransduction. At 24 h, a phosphorylation network centered on CAV1, CTNNB1, and LMNB1 was consistent with Rho GTPase-related structural adaptation. Collectively, these findings provide a hypothesis-generating molecular framework for FIR responses in adult arterial endothelial cells. Functional assays, targeted molecular validation, and dose–response studies are required to determine whether these molecular changes translate into improved endothelial function or vascular repair.

## Data Availability

The original contributions presented in this study are included in the article/Supplementary Material. Further inquiries can be directed to the corresponding author(s).

## Supplementary Materials

The following supporting information can be downloaded at: https://www.mdpi.com/article/10.3390/cells15151390/s1, Table S1: Differentially expressed genes identified in HFAECs following FIR irradiation. Table S2: Differentially expressed proteins identified in HFAECs following FIR irradiation. Table S3: Differentially phosphorylated sites identified in HFAECs following FIR irradiation. Table S4: Pathway enrichment analysis of transcriptomic, proteomic, and phosphoproteomic datasets. Table S5: Angiogenesis-related genes, proteins, and phosphoproteins identified in HFAECs following FIR irradiation. Figure S1: Representative phase-contrast images of HFAECs before and after FIR irradiation.
